# The critical role of the Hippo signaling pathway in kidney diseases

**DOI:** 10.3389/fphar.2022.988175

**Published:** 2022-11-22

**Authors:** Yuting Sun, De Jin, Ziwei Zhang, Di Jin, JiaoJiao Xue, LiYun Duan, YuQing Zhang, XiaoMin Kang, FengMei Lian

**Affiliations:** ^1^ Guang’anmen Hospital, China Academy of Chinese Medical Sciences, Beijing, China; ^2^ Hangzhou Hospital of Traditional Chinese Medicine, Hangzhou, China; ^3^ College of Chinese Medicine, Changchun University of Chinese Medicine, Jilin, China

**Keywords:** hippo, kidney cancer, cystic kidney disease, diabetic kidney diseases, acute kidney injury, chronic kidney injury, renal fibrosis

## Abstract

The Hippo signaling pathway is involved in cell growth, proliferation, and apoptosis, and it plays a key role in regulating organ size, tissue regeneration, and tumor development. The Hippo signaling pathway also participates in the occurrence and development of various human diseases. Recently, many studies have shown that the Hippo pathway is closely related to renal diseases, including renal cancer, cystic kidney disease, diabetic nephropathy, and renal fibrosis, and it promotes the transformation of acute kidney disease to chronic kidney disease (CKD). The present paper summarizes and analyzes the research status of the Hippo signaling pathway in different kidney diseases, and it also summarizes the expression of Hippo signaling pathway components in pathological tissues of kidney diseases. In addition, the present paper discusses the positive therapeutic significance of traditional Chinese medicine (TCM) in regulating the Hippo signaling pathway for treating kidney diseases. This article introduces new targets and ideas for drug development, clinical diagnosis, and treatment of kidney diseases.

## Introduction

In *Drosophila melanogaster*, the Hippo pathway was first identified 20 years ago during tissue growth screening (Xu and Rubin, 1993), and the pathway is conserved between different species. It is also known as the “Hippo pathway” because it makes the mutant fly’s head look like a hippo. The key molecules in the Hippo signaling pathway obtained through chimeric genetic screening in *Drosophila* have corresponding orthologous genes in higher animals, especially mammals. The mutation of the gene encoding the key effector causes tissue overgrowth, which determines the tumor inhibition effect of this pathway (Justice et al., 1995; Kango-Singh et al., 2002; Jia et al., 2003). Early genetic studies on the Hippo pathway showed its role in organ size control (Ma et al., 2019b). During the entire process of vertebrate evolution, replication events have led to evidence of various components of the Hippo family (Cunningham and Hansen, 2022). The Hippo pathway is a highly evolutionarily conserved pathway at the protein kinase level. In the Hippo signaling pathway, the upstream membrane protein receptor functions as a receptor for the extracellular growth inhibition signal. When the extracellular growth inhibition signal is perceived, it activates a series of kinase cascade phosphorylation reactions, leading to phosphorylation of the downstream effectors, yes-associated protein (YAP) and transcriptional coactivator with PDZ-binding motif [TAZ; also called WW domain-containing transcription regulator 1 (WWTR1)]. Several cytoskeleton proteins bind to phosphorylated YAP and TAZ, preventing them from entering the nucleus, which reduces their nuclear activity, ultimately regulating organ size and volume. Recent studies have shown that the Hippo signaling pathway is composed of three components as follows: upstream regulatory module, core protein module, and downstream effector module (Ma et al., 2019b; Moya and Halder, 2019; Pan et al., 2022). The main factors that regulate the Hippo pathway include extracellular matrix (ECM) stiffness, G protein-coupled receptors (GPCRs), cell polarity, and energy stress (Meng et al., 2016). The core protein module is comprised of STE20-like serine/threonine kinase 1/2 (MST1/2) and large tumor suppressor protein 1/2 serine kinase (LATS1/2). The downstream transcription module is mainly composed of YAP and TAZ (Tapon et al., 2002; Harvey et al., 2003; Wu et al., 2003; Zhao et al., 2007; Meng et al., 2016). TAO kinases are capable of activating the Hippo pathway (Meng et al., 2016). Once the upstream signal molecule is activated, MST1/2 and LATS1/2 are phosphorylated, promoting the phosphorylation of YAP and TAZ. In the cytoplasm, phosphorylated YAP and TAZ bind to 14-3-3 proteins, which causes them to be degraded by ubiquitin-dependent proteasomes (Meng et al., 2016). Once the Hippo pathway is inhibited, YAP and TAZ phosphorylation is inhibited, and YAP/TAZ migrates to the nucleus. Yap/TAZ then binds to transcription factors, such as TEA domain transcription factor (TEAD), to regulate cell proliferation (Lei et al., 2019). TEAD family transcription factors are required to mediate the expression of YAP-dependent genes, and TEAD is also necessary for YAP-induced cell growth, epithelial-mesenchymal transformation (EMT), and tumorigenic transformation (Zhao et al., 2008). YAP and TAZ also bind to other transcription factors, including transcription factor 7-like 2 (TCF)/lymphoid enhancer factor (LEF), Smad1, Smad2/3, and p37. Connective tissue growth factor (CTGF) is an important gene for cell growth and is a direct YAP target gene (Zhao et al., 2008; Anorga et al., 2018). CTGF contributes to the regulation of cell growth, proliferation, and apoptosis (Edgar, 2006), thus playing a necessary role in regulating organ size, tissue regeneration, tumorigenesis, and tumor development.

**FIGURE 1:**
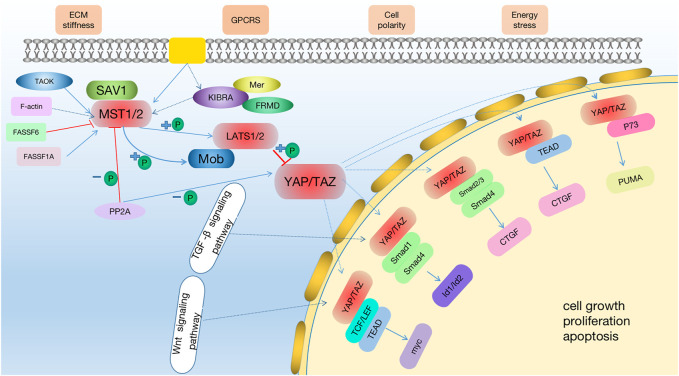
The Hippo signaling pathway. The hippo pathway can be activated by TAO kinase, which phosphorylates MST1/2 in its activation ring. MST1/2 phosphorylated LATS1/2 sequentially with the help of SAV1 and MOB1/2 and then activate YAP/TAZ. Potential treatments: Through the activation of the hippo signaling pathway, MST1/2 and LATS1/2 phosphorylation are activated to improve the level of p-YAP or inhibit the expression of YAP, and then regulate the downstream effector factors to achieve the therapeutic effect on kidney diseases. At the same time, in the early stage of AKI, the expression of YAP is increased, which promotes cell proliferation and promotes the recovery of kidney injury.

Therefore, the research on the molecular function, regulation, and therapeutic targeting of this pathway has become the Frontier in many fields (Dey et al., 2020). Recent studies have shown that the Hippo pathway is intimately related to renal diseases (Wong et al., 2016), including renal cancer, cystic kidney disease, diabetic kidney diseases, and renal fibrosis, and it promotes the transformation of acute kidney disease to chronic kidney disease (CKD). This paper reviews the research status of the Hippo pathway in kidney diseases, further clarifying the function of the Hippo pathway in renal diseases and providing new ideas and a theoretical basis for the treatment of kidney diseases (Figure 1).

## Crosstalk between the Hippo signaling pathway and kidney disease

### Kidney cancer

**FIGURE 2:**
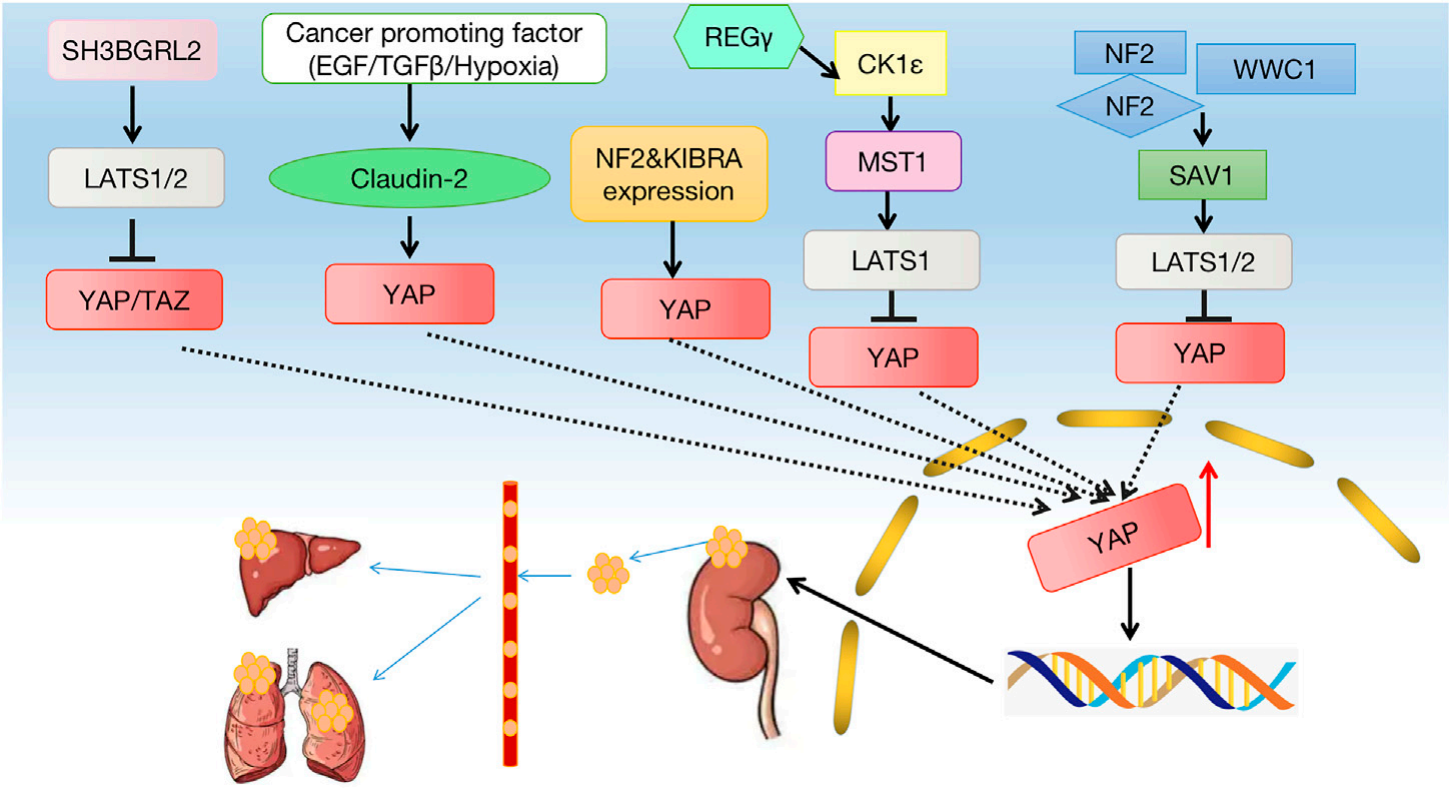
Crosstalk between the Hippo signaling pathway and kidney cancer. Many factors lead to a decrease in phosphorylation of MST1/2 and LATS1/2, which leads to a decrease in phosphorylation of YAP, which leads to a decrease in p-YAP and YAP entering the nucleus. The hippo pathway can affect cell growth, proliferation, apoptosis, and other factors that contribute to the development of kidney cancer.

Among all cancers, 5% are kidney cancers. The highest proportion of renal cell carcinoma (RCC) consists of clear cell renal cell carcinoma (ccRCC), accounting for 70%–80% of kidney cancers (Reuter, 2006). Sheets, cords, and tubes of cancer cells are often present in RCC. Globally, RCC affects nearly 300,000 individuals each year, and the survival rate of RCC depends on the severity of the disease. Patients with local or locally advanced stages of RCC have a 5-year survival rate between 20% and 95%, while patients with metastatic disease have a 5-year survival rate between 0% and 10% (Linehan et al., 2019). Hence, identifying effective therapeutic methods and understanding the mechanism of RCC occurrence and development are urgently needed. According to several studies, RCC is closely associated with the expression of the Hippo pathway. The neurofibromatosis type 2 (NF2) gene, a widely studied tumor suppressor gene, is an upstream regulatory gene of the Hippo pathway, and mutation of NF2 leads to a nonmalignant brain tumor syndrome called neurofibromatosis 2 (Yu et al., 2015; Dodson et al., 2019). Many studies have reported that NF2 enhances the Hippo signaling pathway through phosphorylation, isolation, degradation, and inhibition of YAP/TAZ nuclear translocation, and abnormal Hippo signaling pathway and YAP activation occurs in NF2-deficient unclassified renal cell carcinoma (uRCC) cases (Chen et al., 2016b). Several sporadic cancers, including kidney cancer, are caused by Merlin/NF2, which results in NF2 syndrome, an inherited tumor syndrome (Petrilli and Fernández-Valle, 2016). Silencing YAP/TAZ in NF2-deficient tumors promotes tumor regression (White et al., 2019). Mechanistically, YAP/TAZ depletion increases mitochondrial respiration and reactivity as well as reducing glycolic-dependent growth and causing accumulation of reactive oxygen species (ROS) under nutritional stress, leading to oxidative stress-induced cell death (White et al., 2019). In many types of cancer, LATS1, the core serine/threonine kinase of the Hippo pathway, is reduced. Several mechanisms have been suggested by recent studies to show that LATS1, as a tumor suppressor, negatively regulates tumors and metastases. RCC patients with high expression of LATS1 or LATS2 have significantly longer overall survival (OS) and disease-free survival (DFS) than those with low expression of LATS1/2 (Zhang et al., 2020). Furthermore, RCC tissues and cells express significantly lower levels of LATS1, and renal cell lines express increased levels of hypermethylated LATS1. Pharmacological demethylation of LATS1 with 5-Aza-2′-deoxycytidine (5-AZA) downregulates the expression of YAP, promotes cell apoptosis, promotes cell cycle G1 arrest, and inhibits cell proliferation (Chen et al., 2014). The Yap/TAZ nuclear effector is a gene transcription coactivator that regulates tissue growth and development as part of the Hippo pathway (Rybarczyk et al., 2017). The association between the YAP/TAZ nuclear effector and renal cancer has been demonstrated in many studies. A significant increase in TAZ expression has been found in patients with ccRCC, and the prognosis for these patients is poor (Rybarczyk et al., 2017). TAZ deletion counteracts ferroptosis, which regulates cell death (Yang et al., 2019b). In addition, the expression of epithelial membrane protein1 (EMP1) is regulated by TAZ, which induces the expression of nicotinamide adenine dinucleotide phosphate (NADPH) oxidase 4 (NOX4), a renal-enriched reactive oxygen species (ROS), which produces an enzyme important for ferroptosis (Gorin et al., 2005; Sedeek et al., 2013). In addition, EMP1-NOX4 is regulated by cell density *via* TAZ regulation. Furthermore, the Hippo signaling pathway also plays a role in some key genes related to renal cancer pathogenesis (Yang et al., 2019a). Other research has shown that transferrin (TF) and beta-1,4-N-acetyl-galactosaminyltransferase 1 (B4GALNT1) are highly expressed in patients with ccRCC, and B4GALNT1 may affect the occurrence and progression of renal cancer through the Hippo signaling pathway (Yang et al., 2019a). The Hippo signaling pathway is also important in certain types of kidney cancer. High-grade ccRCC patients still have poor clinical outcomes (Lane et al., 2007). Studies have shown that human Salvador homolog 1 (SAV1), as a component of the Hippo signaling pathway, is downregulated in high-grade ccRCC, which is known to be a tumor suppressor in *Drosophila* (Tapon et al., 2002). In high-grade ccRCC, downregulation of SAV1 and subsequent activation of YAP play a role in the pathogenesis (Matsuura et al., 2011). Mucinous renal tubular spindle cell carcinoma (MTSCC) is a relatively rare renal cell subtype. Several clinical studies have suggested that the Hippo signaling pathway plays a crucial role in the pathogenesis of MTSCC with an enhanced expression of YAP1 being one of the main factors. The use of YAP inhibitors, such as Verteporfin (VP), has also been demonstrated to be effective in treating patients with rare MTSCC with sarcomatoid differentiation or metastatic disease (Mehra et al., 2016). Moreover, the Hippo signaling pathway plays a key role in regulating RCC. Leukemia inhibitory factor receptor (LIFR) plays an important role in the signal transduction of interleukin-6 cytokines (Kishimoto et al., 1995), and inhibition of LIFR kinase activity may be possible because the Hippo pathway is a potential downstream target of LIFR (Chen et al., 2012a). *In vitro* studies have shown that LIFR knockdown leads to decreased levels of LATS1 and p-YAP, resulting in inhibition of the Hippo signaling pathway kinase activity, thereby further increasing YAP expression (Lei et al., 2018). By inhibiting the expression of YAP, LIFR inhibits tumor metastasis. Taurine upregulated gene 1 (TUG1) is involved in the regulation of RCC as TUG1 positively regulates YAP by downregulating the expression of microRNA-9 (miR-9), thus regulating the growth and migration of RCC (Liu et al., 2018). Claudin-2 inhibition induces mesenchymal plasticity and invasive mobility. In RCC-derived cancer cells, overexpression of Claudin-2 inhibits tumorigenesis and tumor growth in xenografts (Kumar et al., 2021). Claudin-2 binds to YAP through its PDZ motif [a member of the mitogen-activated protein kinase kinase (MAPKK) family] and regulates its nuclear activation and expression. SH3 domain binding glutamate-rich protein-like 2 (SH3BGRL2) is critical for EMT progression and metastasis in ccRCC, and SH3BGRL2 exerts tumor inhibition through the Hippo pathway (Yin et al., 2020). Quaking RNA-binding protein (QKI) regulates angiogenesis, embryogenesis, glial cell differentiation, apoptosis, and transcription, and it also regulates tumor cell metabolism, differentiation, proliferation, and immunity (Noveroske et al., 2002; Wu et al., 2002; Lee and Schedl, 2004; Larocque et al., 2005). Immunohistochemistry of clinical specimens has demonstrated a positive correlation between QKI and p-YAP. YAP is negatively regulated by QKI, which regulates cell contact inhibition and inhibits proliferation and invasion of tumor cells *via* Wnt and GPCR signaling pathways (Zhu et al., 2019). Moreover, REGγ depletion activates the Hippo signaling pathway by stabilizing casein kinase 1ε (CK1ε), suggesting crosstalk between the REGγ/CK1ε axis and the Hippo pathway in RCC (Chen et al., 2018b). KIBRA, a human tumor suppressor protein expressed in the kidney and brain, has a role in regulating cell contact inhibition, tissue regeneration, organ size, and tumor development and progression. KIBRA also plays a key role in tumor progression and metastasis. In clear cell carcinomas, KIBRA is epigenetically downregulated (Schelleckes et al., 2017). The relationship between the Hippo signaling pathway and some tumor-related factors confirms the close relationship between the Hippo signaling pathway and renal cancer, providing ideas for the treatment of renal cancer (Figure 2).

### Cystic kidney disease

**FIGURE 3:**
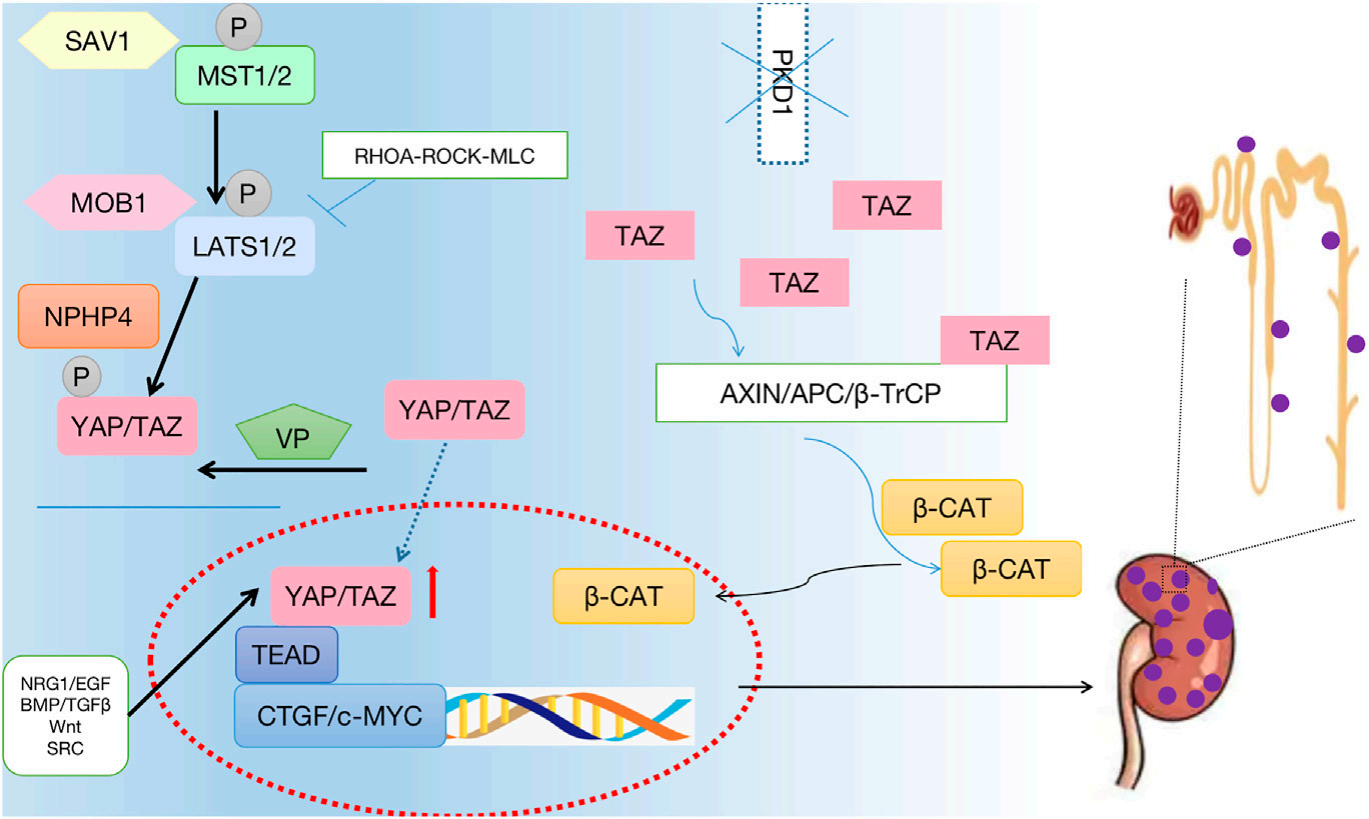
Crosstalk between the Hippo signaling pathway and the polycystic kidney disease. The phosphorylation of core protein MST1/2 and LATS1/2 was inhibited, and the phosphorylation level of downstream effector factor YAP was inhibited. YAP enters the nucleus, hippo pathway affects cell growth, proliferation, apoptosis. Thus, promoting the occurrence of polycystic kidney.

The two main forms of monogenic polycystic kidney disease are autosomal dominant polycystic kidney disease (ADPKD) and autosomal recessive polycystic kidney disease (ARPKD), which are cilia-related diseases (Müller and Schermer, 2020). ADPKD is the most common monogene genetic system disease in humans (Bergmann et al., 2018), and it is caused by genetic mutations in PKD1 [encoding polycystin-1 (PC1)] or PKD2 [encoding polycystin-2 (PC2)] (Chapin and Caplan, 2010). ADPKD is characterized by progressive deterioration of renal function and spontaneous formation of fluid-filled renal cysts, which cause end-stage renal disease (ESRD) (Cornec-Le Gall et al., 2019). Compared to wild-type rats, the expression of LATS1 in cyst-lining epithelial cells of Han:SPRD heterozygous rats decreases, and the expression of YAP and dephosphorylation activation levels increase. Moreover, PKD1 mutations have a functional link with the Hippo signaling pathway (Cai et al., 2018). Four-jointed (Fj) regulates the Hippo signaling pathway through a complex mechanism (Willecke et al., 2008). The levels of PKD1 and PKD2 genes are associated with normal kidney development, and overexpression of these genes leads to renal cystic disease phenotypes (Thivierge et al., 2006; Park et al., 2009; Kurbegovic et al., 2010). Tubule epithelial damage, such as induced cyst formation, occurs in Pkd1 mutant mice, and the expression of YAP, a downstream factor of the Hippo signaling pathway, in the cytoplasm repairs the tissue. In advanced cystic epithelium and dilated tubule epithelium, YAP accumulates in the nucleus, accompanied by upregulation of the YAP transcription targets, baculoviral IAP repeat-containing 3 (BIRC-3), inhibin beta-A (INHBA), four-jointed box kinase 1 (Fjx1), and CTGF. A change in the Hippo pathway activity has been replicated in human renal tissue from patients with ADPKD and ARPKD with cystic renal tumors (Happé et al., 2011). The Pkd1^−/−^ cell transcriptome and pre-cystic kidney transcriptome indicate the involvement of the Hippo signaling pathway in early polycystic kidney disease. *In vitro* and *in vivo* Pkd1del models show changes in MAPK, Wnt, Hippo, PI3K/Akt, and calcium signaling pathways related to ADPKD (Kunnen et al., 2018). At the same time, many pathogenic genes affecting cystic kidney disease act through the Hippo signaling pathway. FAT1 has been demonstrated to be an effective regulator of Hippo signals (Willecke et al., 2006; Katoh, 2012; Ahmed et al., 2015; Martin et al., 2018), and deletion of FAT1 leads to the formation of zebrafish anterior kidney cysts (Skouloudaki et al., 2009). NIMA-related kinase 8 (NEK8) acts as a regulatory factor affecting the Hippo signaling pathway (Habbig et al., 2012). Treatment of NEK8 knockout morphogenetic defective zebrafish embryos or epithelial cells with VP rescues the phenotype, indicating that YAP plays a therapeutic role in the occurrence of cystic kidney disease (Grampa et al., 2016). NEKs regulate cilia and cell cycle progression, and NEK8 localizes to the centrosome and the proximal region of cilia. The non-catalytic RCC1 motif of NEK8 comprises the C-terminal activity and kinase structure (Shiba et al., 2010). Sorting nexin 9 (SNX9) inhibits cell proliferation and cyst development in ADPKD by activating Hippo signaling (Shen et al., 2020). A member of the MAGUK p55 family, PALS1, mediates the connection between TGF-β and Hippo signaling pathways, and it participates in cyst formation. PALS1 is expressed in the human renal epithelium as a core polar protein (Weide et al., 2017). The TAZ/Wnt-β-catenin/c-MYC axis regulates the Hippo signaling pathway of polycystic kidney disease (Lee et al., 2020). TAZ induces Wnt/TGF-β signal target gene overlap and c-Myc mRNA expression (Choi et al., 2018). In Pkd1-deficient mice, TAZ is expressed around the inner epithelial cells of renal cysts, and TAZ deletion reduces cyst formation. In wild-type mice, TAZ interacts with PKD1 to inactivate β-catenin. In Pkd1-deficient cells, TAZ interacts with axin1 to increase β-catenin activity. TAZ is one of the upstream regulators of c-Myc expression (Harris and Torres, 2009). RhoA/YAP/c-Myc signaling plays a crucial role in ADPKD pathogenesis caused by Pkd1 deficiency (Ma and Guan, 2018). Several studies have reported that the RhoA/YAP/c-Myc signal axis promotes polycystic kidney disease development and occurrence (Cai et al., 2018). A new strategy to treat cystic kidney disease may be derived from the Hippo signaling pathway as it mediates the pathogenesis of cystic kidney disease (Figure 3).

### Acute kidney injury

Worldwide, AKI has a high mortality rate (over 50%) (Zhou et al., 2020) with an approximate 15%–20% incidence of AKI in hospitalized patients and an approximate 50%–70% incidence of AKI in intensive care unit (ICU) patients (Makris and Spanou, 2016). The Hippo signaling pathway and AKI have been linked in a recent study. AKI is generally regarded as an independent risk factor for progression to CKD, and patients with AKI requiring dialysis have a greater probability of developing CKD and ESRD (Chawla et al., 2011). There is a close association between YAP and AKI, and it is a downstream effector of the Hippo signaling pathway. Xu et aldetected the expression of the Hippo signaling pathway components in the kidney of a rat model of AKI and renal biopsy specimens of patients with AKI; these researchers found that YAP protein levels increase in the cytoplasm and nucleus of renal tubular epithelial cells (TECs) during the AKI repair stage, and they reported that the change of YAP expression is positively correlated with the change of TEAD expression, a YAP downstream target (Xu et al., 2016). Additionally, AKI is positively correlated with CTGF expression, which plays a crucial role in renal fibrosis development and occurrence. Furthermore, apoptosis plays an important role as an early manifestation of injured cells after ischemia-reperfusion (IR), and apoptosis is also linked to AKI. The NF2 signaling pathway likely regulates the apoptosis of IR tissue cells through the Hippo pathway (Grannas et al., 2015). In addition, renal tubular epithelial EMT is closely related to AKI. Renal tubular epithelial EMT is a process in which renal TECs lose their epithelial phenotype of cell polarity and cell adhesion, acquiring the migration and invasion abilities unique to mesenchymal stem cells (Yang and Liu, 2001). EMT occurs in tubule epithelial cells when their polarity is lost and TGF-β/Smad signaling is activated. Cell polarity is maintained by the Crumbs (CRB)/PALS1 complex, which regulates the Hippo signaling pathway (Iwakura et al., 2017). The Hippo signaling pathway may be related to AKI because cell resistance is limited to the complete recovery period of proximal tubule (PT) cells, such as occurs in PT cells in experimental rats treated with uranium after AKI recovery, producing cells resistant to subsequent UA treatment. The presence of PT cells after AKI recovery may be due to YAP-mediated inhibition of apoptosis, resulting in acquired resistance in cells; however, this effect does not occur until PT remodeling is complete, and the number of cells is normal (Formica et al., 2019). The EGF receptor (EGFR) is widely expressed in mammalian kidneys, particularly in renal proximal tubule epithelial cells (RPTCs) (Breyer et al., 1990; Chen et al., 2012b). EGFR-dependent YAP activation is key for AKI renal recovery, and there is increasing evidence that renal recovery is due to the differentiation and proliferation of surviving TECs (Chen et al., 2018a). Chen et alfound that the expression of YAP increases in RPTCs of AKI patients and mice, inhibiting the related effects of the YAP/TEAD transcription factor complex through VP, and they reported that deletion of YAP from RPTCs delays the recovery of renal function and structure from IR injury (IRI). Thus, studying the Hippo pathway in AKI research may be a promising prospect.

### Chronic kidney disease

CKD continues to remain a major public health burden (Perico and Remuzzi, 2012). Podocytes are responsible for setting up a hematuria filtration barrier in kidney glomeruli (Bonse et al., 2018) and are essential for maintaining kidney function (Hurcombe et al., 2019). The result of persistent podocyte damage is podocyte loss, which eventually leads to ESRD. The localization of YAP is an important regulator of podocyte function, and apoptosis is induced by various treatments (Bonse et al., 2018). Sequence analysis of LATS2-overexpressing podocytes has demonstrated that apoptotic genes are significantly induced. Downregulation of Hippo signaling pathway components results in a feedback mechanism in podocytes. The link among podocytes, Hippo signaling pathway activation, and *in vivo* regulation of connection and migration processes is the basic mechanism of glomerulosclerosis and renal function loss. Rac1, a member of the Rho family of GTPases, plays an important role in cell movement, cell bone remodeling, and cell cycle transport (Jaffe and Hall, 2005). Rac1 GTPases promote TGF-β signaling through the EGFR, Hippo/YAP/TAZ, and p53 pathways to regulate fibrosis and CKD (Patel et al., 2019). Patients with FAT1 mutations present with ophthalmic disease with changes from normal renal function to early-onset end-stage kidney failure (Fabretti et al., 2021). Therefore, elucidating the precise mechanism of the Hippo signaling pathway in CKD will enrich the pathogenesis theory of CKD and provide a new method for clinical targeted diagnosis, treatment, and prevention of CKD.

### Diabetic kidney disease

**FIGURE 4:**
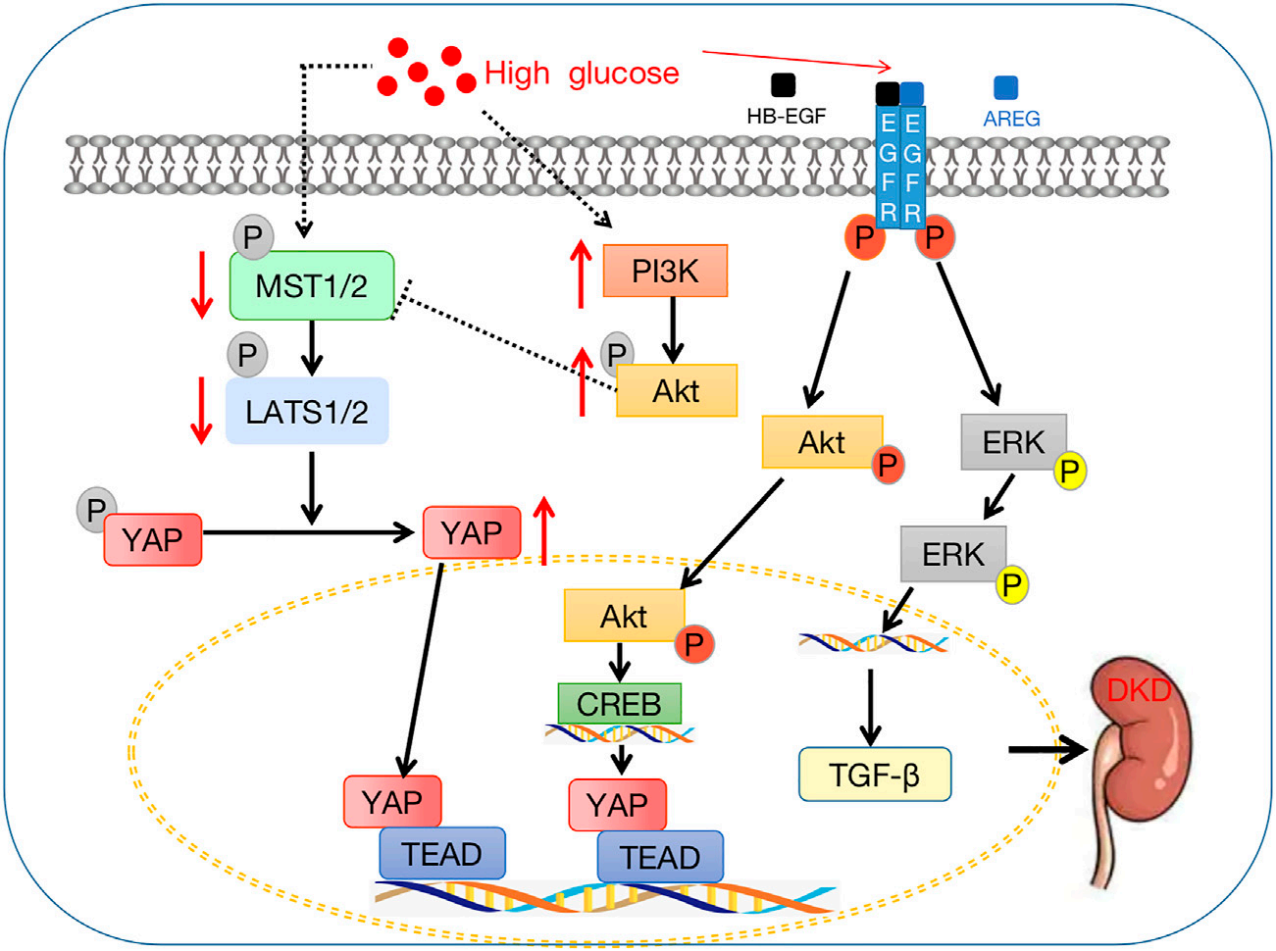
Crosstalk between the Hippo signaling pathway and diabetic kidney disease. The downstream effector factor YAP enters the nucleus, binds with TEAD, and then affects the downstream target gene CTGF to promote the occurrence and development of DKD.

A common microvascular complication of diabetes is diabetic kidney disease (DKD). Approximately 30% of patients with type 1 diabetes and 20%–50% of patients with type 2 diabetes may develop DKD (Lin et al., 2018). Thus, studying DKD pathogenesis and controlling its progression have become urgent tasks. For the occurrence and progression of DKD, targeting the Hippo signaling pathway may be a therapeutic strategy. YAP, a downstream effector of the Hippo signaling pathway, is closely related to DKD. Diabetes-related renal interstitial fibrosis (RIF) is promoted by YAP based on its ability to activate epithelial interstitial transformation. Furthermore, RPTC-specific YAP deficiency or treatment with the YAP inhibitor, VP, significantly reduces diabetic RIF (Chen et al., 2020). Activation of YAP promotes its interaction with TEAD, which contains DNA, and the YAP/TEAD complex then activates the overexpression of these target genes, promoting cell proliferation and ECM synthesis (Breyer et al., 1990). TEAD is a factor with a DNA-binding domain that binds to activated YAP to control the expression of its important target protein factor, CTGF (Zhao et al., 2010). There is a higher concentration of YAP, TEAD, and CTGF in the nuclei of glomerular cells of patients with type 2 DKD. The high expression of YAP, TEAD, and CTGF in renal tissue suggests that YAP plays a key role in renal damage in type 2 diabetes mellitus (Ma et al., 2019b). The high expression of YAP is associated with the increase in systolic blood pressure (SBP), blood urea nitrogen (BUN), and creatinine (Cr) as well as the progression of DKD staging and pathological classification of DKD (Ma et al., 2019b). Inhibition of YAP activity may delay the progression of DKD. MST1, as the core protein module of the Hippo signaling pathway, is involved in cell proliferation and differentiation, and it plays a key role in DKD. Targeting MST1 may be a potential therapeutic target for DKD. YAP/TEAD-mediated EMT ameliorates DKD fibrosis by targeting MST1. MST1 activation is reduced in type 1 and type 2 DKD. In HK-2 cells, MST1 is downregulated in a glucose- and time-dependent manner (Yang et al., 2020). Mst1 downregulation promotes renal dysfunction and fibrosis in db/m mice *in vivo*. Moreover, MST1 inhibits the activation of YAP by binding to TEAD to form a YAP/TEAD heterodimer, which directly regulates TEAD activation, thereby stimulating EMT. The Hippo signaling pathway is also closely related to other related factors, thus affecting DKD. EGFR regulation of the Hippo signaling pathway is an important mechanism for the occurrence and development of DKD (Reddy and Irvine, 2013). Many experiments have found that EGFR and its different ligands are upregulated and that EGFR activation occurs in renal cells cultured in a high glucose environment and in experimental diabetic models (kidney injury) (Miyazawa et al., 2013). According to previous studies, erlotinib, an EGFR kinase inhibitor, and EGFRptko mice inhibit the expression and phosphorylation of YAP protein. In addition, activation of the EGFR/PI3k/Akt/CREB signaling pathway mediates YAP gene expression, YAP nuclear expression, and interaction with TEAD transcription factor complexes, resulting in the upregulation of one of two TEAD-dependent genes, namely, CTGF and two-way regulatory genes. Pharmacological or genetic inhibition of YAP by EGFR, Akt, or CREB improves DKD in proximal renal tubular cell lines (Chen and Harris, 2016). The proliferation of mesangial cells (MCs) is regulated by YAP-mediated PI3K/Akt and Hippo signaling pathways. Protein kinase B (PKB, also known as Akt), which controls protein synthesis, cell development, and proliferation, is phosphorylated by phosphoinositol 3-kinase (PI3K) (García et al., 2006; Kim et al., 2012). High glucose-treated mice and MCs show decreased phosphorylation levels of MST1 and LATS1 but increased PI3K/Akt activation and proliferation (Qian et al., 2021). YAP activates the PI3K/Akt pathway by inhibiting PTEN, a repressor of PI3K. When the Hippo signaling pathway is inhibited and PI3K/Akt signaling is activated, YAP accumulates in the nucleus and promotes MC proliferation and DKD formation. At the same time, activation of the PI3K signaling pathway inhibits the phosphorylation of MST1 and LATS1 as well as accelerating YAP activation and transport to the nucleus, thereby promoting the transcriptional role of YAP (Collak et al., 2012; Fan et al., 2013). In addition, the activation of YAP inhibits PTEN and further activates the PI3K/Akt pathway, forming a positive feedback loop of YAP→PTEN→PI3K/Akt→MST1→LATS1→YAP (Qian et al., 2021). The existence of this positive feedback mechanism confirms that inhibiting the activity of the Hippo signaling pathway promotes the increase of MCs and accelerates the occurrence and development of DKD. It has been demonstrated that the Hippo signaling pathway contributes to the regulation of renal pathophysiology at the multicellular level in DKD pathogenesis, thereby affecting renal function (Lei et al., 2019) (Figure 4).

## Crosstalk between renal fibrosis and the Hippo signaling pathway

Renal fibrosis is the common pathway from secondary progressive CKD to ESRD, and renal fibrosis is a major determinant of renal insufficiency (Liu, 2011). In DKD, EMT plays an important role in the apoptosis pathogenesis of RIF (Inoue et al., 2015; Lovisa et al., 2016; Zhao et al., 2017). A process in which endothelial cells lose adhesion, lose polarity, and become spindle mesenchymal cells, which are highly invasive and metastatic, occurs due to this activity. When biomechanical properties of tissues change, the YAP/TAZ sensor is activated, which causes pro-inflammatory and pro-fibrogenic signals to be released (Landolt et al., 2022). After ischemic AKI, YAP is a key influencing factor of regeneration and fibrosis (Xu et al., 2016). Fibrosis of the kidney has been linked to aberrant YAP activation in an increasing number of studies (Szeto et al., 2016; McNeill and Reginensi, 2017; Kim et al., 2019). Because continuous elevation and activation of YAP are associated with interstitial fibrosis and abnormal renal tubule differentiation, appropriate regulation of YAP protein is an effective therapeutic target during AKI-CKD transformation after IRI. Wnt5a promotes renal fibrosis by stimulating the M2 polarization of macrophages mediated by YAP/TAZ (Park et al., 2015). Many studies have reported the interaction between the Hippo signaling pathway and the TGF-β signaling pathway. For example, TGF-β increases the response to the TGF-signaling pathway by stimulating TAZ to bind to the active Smad complex in the nucleus (Varelas et al., 2008; Wrighton et al., 2008). Feng found that the Wnt5a signaling protein enhances the M2 polarization of macrophages induced by transforming growth factor β1 (TGF-β1) and the expression of YAP/TAZ. VP blockade of YAP/TAZ inhibits the M2 polarization of macrophages induced by the Wnt5a and TGF-β1 pathways (Feng et al., 2018). Src, a proto-oncogene tyrosine-protein kinase, is a non-receptor tyrosine kinase, and the Src-mediated association between FXR and YAP protects against renal fibrosis. The Farnesoid X-activated receptor [FXR, which is also known as nuclear receptor subfamily 1 group member 4 (NR1H4)] is a multifunctional transcription factor that plays a key role in the prevention of fibrosis (Fiorucci et al., 2004; Wang et al., 2008; Modica et al., 2010; Calkin and Tontonoz, 2012; Kim et al., 2015). The phosphorylation and nuclear localization of YAP is regulated by GW4064-mediated FXR activation and inhibition of Src activation in renal fibrosis. GW4064 and the Hippo signaling pathway core kinases (MST1 and LATS1) induce phosphorylation of YAP, causing cytoplasmic accumulation of YAP. Inhibition of Src with PP2 (Src kinase inhibitor) prevents renal fibrosis, increases p-YAP phosphorylation, and increases YAP cytoplasmic accumulation. The use of GW4064 or WAY-362450 (turofexorate isopropyl) also prevents unilateral ureteral obstruction-induced renal fibrosis (Kim et al., 2019). Renal fibrosis is affected by the Hippo signaling pathway in conjunction with other related pathways.

**FIGURE 5:**
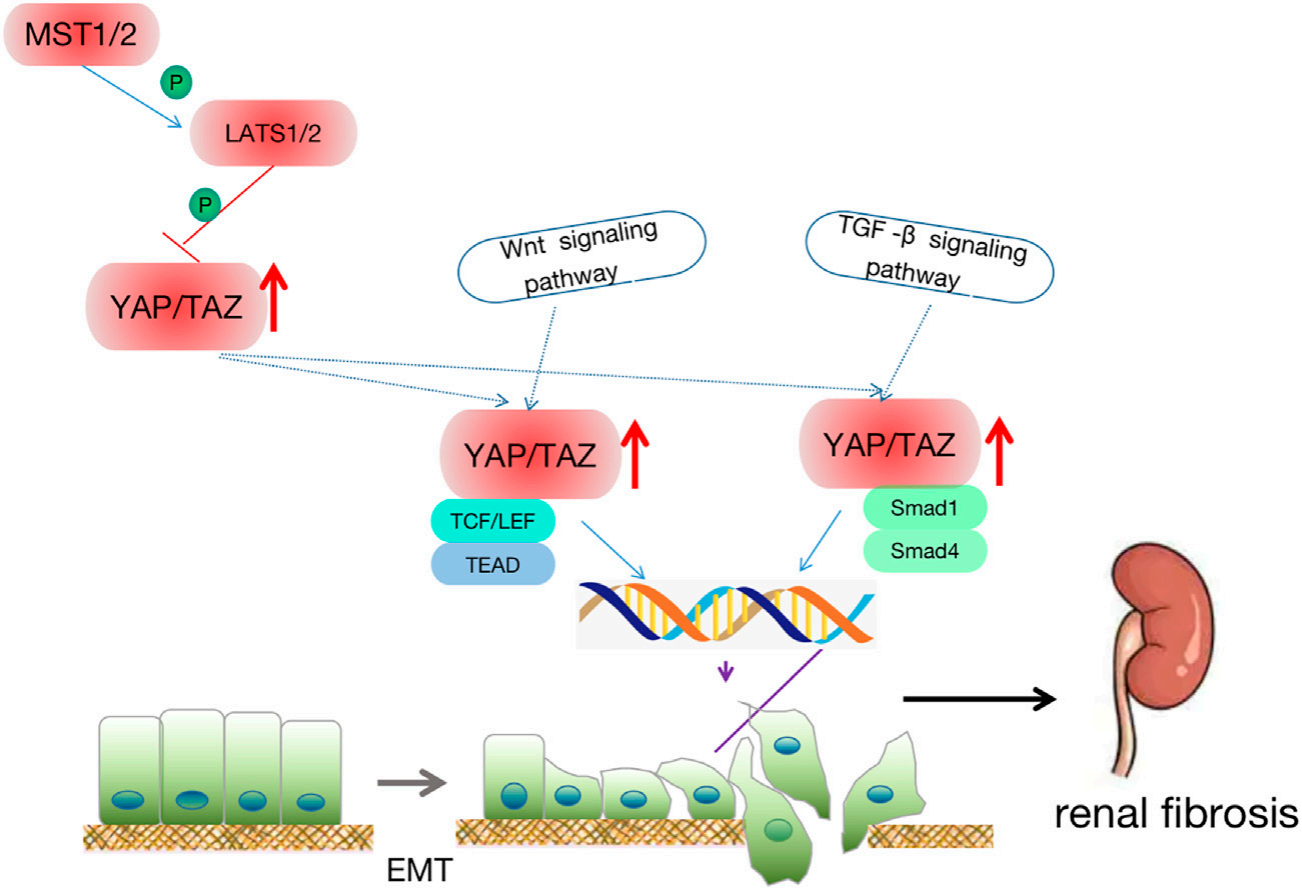
Crosstalk between renal fibrosis and the Hippo signaling pathway. Downstream effector YAP enters the nucleus and binds to TEAD, Smad1/Smad4 to promote epithelial-mesenchymal transition. At the same time, the hippo signaling pathway, TGF, Wnt, and other signaling pathways interact together to participate in cell apoptosis, regeneration, and EMT, thus leading to renal fibrosis.

The Hippo-Salvador signaling pathway regulates renal tubule interstitial fibrosis. In Salvador mice with specific loss of TECs after unilateral ureteral obstruction (UUO), the Hippo-Salvador signaling pathway increases renal tubule interstitial fibrosis (Seo et al., 2016). In addition, the EMT phenotype changes are enhanced, and apoptosis and proliferation are observed. TECs increase the expression of TGF-β and activate the expression of β-catenin after depletion of UUO by Sav1. In addition, TAZ is significantly activated in SAV1 knockout mice, and TAZ directly regulates TGF-β and TGF-β receptor II expression. Hepatocyte growth factor (HGF) induces EMT by regulating the MST2 and ISG15 signaling pathways. An enrichment analysis has suggested that ubiquitination-related proteins and apoptosis-related proteins are induced, whereas proteins regulating apoptosis are inhibited. ITCH, an E3 ubiquitin ligase, ubiquitinates the LATS1 tumor suppressor, leading to degradation (Ho et al., 2011; Salah et al., 2011), and the ITCH ubiquitin ligase regulates mammalian MST2 and ISG15 pathways at the protein level. Inhibition of the Hippo signaling pathway may accelerate HGF-induced EMT by overexpression of ITCH or A-Raf (Farrell et al., 2014). The Hippo pathway-related factors affect renal fibrosis by regulating the Hippo signaling pathway. Krüppel-like factor4 (KLF4) is a bifunctional transcription factor that activates or inhibits the transcription of genes that regulate cell proliferation and differentiation (Brauer et al., 2018; Cheng et al., 2018; Choi and Roh, 2018; Tang et al., 2018; Qi et al., 2019). As a result of IR renal injury, YAP is continuously activated by KLF4 and promotes renal fibrosis in mice. Previous studies have demonstrated that YAP downregulation significantly reduces IR-induced renal dysfunction as well as reducing the expression of the TGF-β and CTGF renal fibrosis factors. Moreover, the expression of KLF4, a transcription factor upstream of YAP, is also continuously increased, and inhibition of KLF4 reduces YAP elevation, nuclear translocation, renal deterioration, and interstitial fibrosis in IR mice (Xu et al., 2021). The Hippo signaling pathway also affects renal fibrosis through drugs. VP is a photosensitizer used to treat age-related macular degeneration (Chen et al., 2015). VP inhibits members of the TEAD-YAP interaction in the absence of light stimulation in cancer cells (Liu-Chittenden et al., 2012), and inhibition of YAP proteins by VP improves renal tubule interstitial inflammation and fibrosis caused by UUO. In mice, Jin et alfound that VP reduces UUO-induced renal tubule injury and inflammation as well as increasing ECM deposition (Jin et al., 2020). Therefore, it would be advantageous to explore the Hippo signaling pathway as a new target for treating renal fibrosis (Figure 5).

## Expression of Hippo signaling pathway components in renal pathological tissues

**FIGURE 6:**
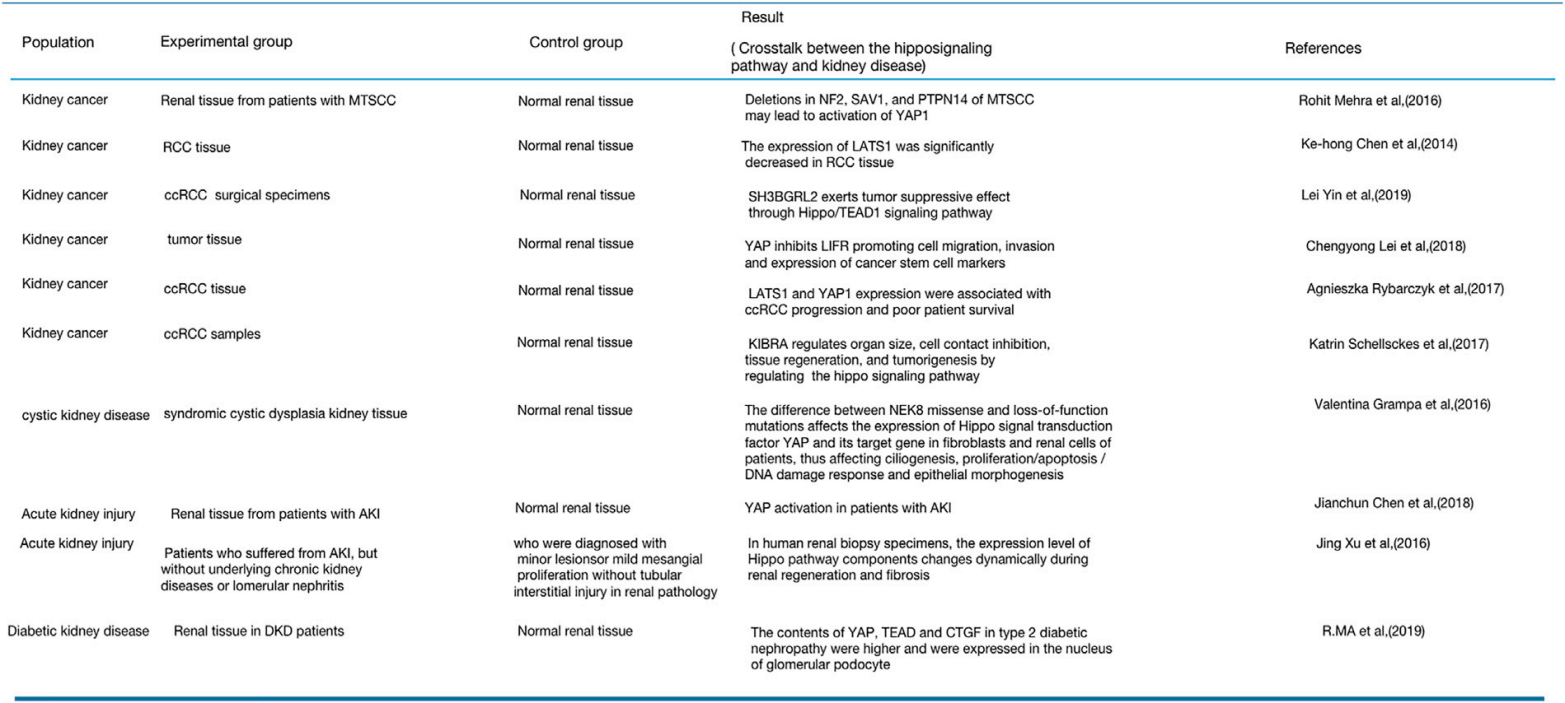
Expression of the Hippo signaling pathway in renal pathological tissues.

The Hippo signaling pathway regulates cell growth, proliferation, and apoptosis as well as organ, tissue, and tumor development (Edgar, 2006). Analysis of many renal pathological tissues has demonstrated that the Hippo pathway also plays a significant role in the progress of kidney diseases. Kidney cancer, cystic kidney disease, and DKD are all related to the Hippo signaling pathway (Chen et al., 2014; Grampa et al., 2016; Mehra et al., 2016; Xu et al., 2016; Rybarczyk et al., 2017; Chen et al., 2018a; Lei et al., 2018; Ma et al., 2019a). Figure 6 summarizes the expression of Hippo signaling pathway components in renal pathological tissues.

## Traditional Chinese medicine regulates kidney diseases by affecting the Hippo signaling pathway

### Triptolide

Triptolide (TP) is a diterpenoid trioxide compound that has anti-inflammatory and immunomodulatory properties (Ma et al., 2019a). TP is clinically used for treating glomerulonephritis, IgA nephropathy, and membranous nephropathy, and its mechanism may be immunosuppressive and anti-inflammatory. Experimental results have shown that TP regulates cell polarity, cell adhesion, and EMT through its effects on TGF-β1 and Hippo signaling pathways (Chen et al., 2010). At the same time, TP inhibits the EMT of renal TECs (Niedermayer et al., 2015), and studies have demonstrated that inhibiting renal tubular EMT effectively inhibits renal fibrosis (Zang, 2019). Treatment of renal TECs with TP results in TAZ levels close to normal levels in cells undergoing EMT. Thus, TP inhibits the EMT of renal tubular epithelium by modulating the Hippo signaling pathway, which reduces renal tubular epithelial fibrosis (Niedermayer et al., 2015).

### Quercetin

Quercetin is a type of biological flavonoid compound, and it has a variety of pharmacological effects, including reduction of the risk of kidney and cardiovascular diseases as well as antitumor, antioxidant, antiviral, and anti-inflammatory effects (Hazzan et al., 2011; Bournival et al., 2012; Lu et al., 2015; Chen et al., 2016a; Vaidya et al., 2016; Sánchez-González et al., 2017; Patel et al., 2018). In recent years, many studies have shown that quercetin inhibits tumors (Vaidya et al., 2016) and promotes cell apoptosis and cell cycle arrest (Chen et al., 2012c). Studies have demonstrated that quercetin also significantly reduces the expression of glycosylation end products, including laminin, type IV collagen, and connective tissue growth factor, which inhibits the proliferation of MCs, and quercetin has also been shown to reduce the thickness of the glomerular basement membrane (Vaidya et al., 2016; Sánchez-González et al., 2017). DKD is characterized by the proliferation of (MCs), a pathological change that occurs at an early stage of the disease. Previous research has found that the phosphorylation levels of MST1 and LATS1 are markedly reduced in high glucose-induced MCs and db/db mice compared to controls, and quercetin reverses these changes in a dose-dependent manner. Quercetin treatment reverses the increase in YAP expression in the high glucose group. According to experimental results, culture of MCs in high glucose inhibits Hippo signaling, whereas quercetin reactivates Hippo signaling to inhibit the proliferation of MCs (Lei et al., 2019).

Although TP and quercetin have contradictory functions in many biological processes, they both regulate kidney disease by modulating the Hippo signaling pathway. Through its ability to regulate the Hippo signaling pathway, TP reduces renal tubular epithelial fibrosis (Niedermayer et al., 2015). The Hippo signaling pathway is regulated by quercetin, which inhibits MC proliferation and improves early DKD symptoms (Ma et al., 2019b). In addition to its role in the apoptosis mechanism of DKD RIF, EMT also plays an important role in tissue fibrosis (Inoue et al., 2015; Lovisa et al., 2016; Zhao et al., 2017). Both EMT and DKD are closely related to renal fibrosis. Thus, both TP and quercetin may slow the progression of kidney disease through the regulation of the Hippo signaling pathway and contribute to renal fibrosis prevention. There is still a lack of clinical evidence to support the application of traditional Chinese medicine in kidney diseases involving Hippo signaling pathway. Therefore, clinical trials are further carried out to promote the clinical application of traditional Chinese medicine (TCM) in regulating hippo signaling pathway. The use of TCM to interfere with the targets of the Hippo pathway may provide ideas for the intervention and treatment of kidney diseases, which is expected to delay the progression of kidney diseases and improve the prognosis.

## Limitations and prospects

Nephropathy research has shown that some nephropathy is closely associated with the Hippo signaling pathway; however, there are still many limitations. At present, it is not completely understood how to modulate the Hippo signaling pathway interaction with metabolism at the cellular level to control systemic metabolism under physiological and pathological conditions, indicating the need for additional studies. It has been reported that renal fibrosis is the result of multiple signaling pathways, such as the Wnt, Notch, and Hippo signaling pathways, and corresponding cytokines, such as TGF-β. Fibrosis of the kidney is a relatively complex process, including signaling pathways interacting with one another. Targeting one signaling pathway or cytokine may not allow the full understanding of the occurrence, development, and prevention of renal fibrosis (Casella et al., 2014). The specific molecular mechanism of the Hippo signaling pathway in AKI-induced renal tubular epithelial cell apoptosis, regeneration, EMT, and interstitial fibrosis as well as its interaction with the Wnt/β-catenin and TGF-β/Smad signaling pathways need further elucidation (Shan, 2021). Therefore, future studies should explore multiple signaling pathways and their cross-correlation to comprehensively understand the process of renal fibrosis and to identify the key intersection between signaling pathways. Additionally, the targeted regulation between the Hippo pathway and cytokines needs to be further investigated (Zheng et al., 2017). Future studies need to explore strategies (such as modulation of YAP, LATS1/2, and MST1/2 expression) that will help make clinical diagnosis more accurate, more sensitive, and faster to allow assessment of the progress and improve the treatment and prognosis of kidney diseases. However, there is no consensus on the best clinical indication, and high-quality clinical trials are still needed to promote its clinical application. Additionally, TCM currently lacks sufficient knowledge of how the Hippo signaling pathway regulates kidney disease. In the future, in-depth research on the regulation of the Hippo signaling pathway by TCM will provide new ideas and targets for the treatment of kidney diseases.

## Conclusion

Recent studies have shown that the Hippo signaling pathway regulates cell proliferation and apoptosis and that it is closely associated with organ structure and function. In addition, the Hippo signaling pathway is involved in the occurrence and development of nephropathy. Recent in-depth studies of signaling pathways have revealed their specificity and diversity in different tissues, cells, and organs. The signaling pathways and molecular mechanisms involved in diseases are complex with various effects resulting from the same molecule occurring in different cells. Thus, the signaling pathways involved in kidney disease need further study to achieve advancements in drug development, clinical diagnosis, and treatment, thereby providing hope for improved treatment of kidney diseases.
